# The effect of propofol on the grading of diastolic function: a prospective observational study

**DOI:** 10.1186/s12871-025-03260-2

**Published:** 2025-07-30

**Authors:** Shalin Desai, Zachary Hamilton, Lynh McCloskey, Orode Badakhsh, David Li, Neal W. Fleming

**Affiliations:** 1 School of Medicine, University of California, Davis, Sacramento, CA USA; 2 Clinical Professor, Department of Anesthesiology & Pain Medicine, University of California, Davis, Sacramento, CA USA; 3 Associate Clinical Professor, Department of Anesthesiology & Pain Medicine, University of California, Davis, Sacramento, CA USA; 4 Professor Emeritus of Clinical Anesthesiology, Department of Anesthesiology & Pain Medicine, University of California, Davis, 4150 V Street Suite 1200 PSSB, Sacramento, CA 95817 USA

**Keywords:** Diastolic function, Propofol, General anesthesia, Tissue doppler imaging, Mitral flow velocity

## Abstract

**Background:**

Left ventricular diastolic dysfunction accounts for approximately half the cases of heart failure in the community. Its strong association with both peri-operative and post-operative adverse outcomes in both non-cardiac and cardiac surgical patients highlights the importance of accurately measuring diastolic function peri-operatively. Previous studies examining general anesthesia and diastolic function have shown varying impacts of propofol, ranging from improvement to no effect or worsening of diastolic function. This study aimed to evaluate the impact of propofol and mechanical ventilation on left ventricular diastolic function assessment by comparing pre-induction to post-induction measurements.

**Methods:**

This was an analysis of a subset of data from a prospective, observational, single-center study. Written informed consent was obtained from patients who were at least 18 years of age and undergoing elective, non-cardiac surgery with planned use of intra-arterial blood pressure monitoring. After routine pre-medication (midazolam, 1-2 mg IV) was administered, diastolic function was assessed using trans-thoracic echocardiography (TTE). The following parameters were obtained: lateral mitral annular tissue doppler velocity (e’), peak early mitral flow velocity (E) and peak late mitral flow velocity (A). Five minutes after induction of anesthesia with propofol and initiation of mechanical ventilation, a repeat TTE was performed. Using the simplified algorithm described by Swaminathan et al., the diastolic measurements were analyzed, and the patient was assigned a grade.

**Results:**

Data for analysis was available from 113 patients. All values are presented as median [95%CI]. There were significant decreases in e’ (9.1 [8.7, 10.5] to 8.2 [7.2, 8.9] cm/s) {*p* < 0.0001}, E (79.8 [76.0, 83.4] to 66.0 [62.8, 72.0] cm/s) {*p* < 0.0001}, and A (82.2 [75.3, 89.0] to 64.1 [60.2, 69.0] cm/s) {*p* < 0.0001}. 49 patients (43%) had a change in their diastolic function grades from pre-induction to post-induction. Of those 49 patients, 20 (41%) had an improvement, while 29 (59%) had a worsening of their diastolic function grade. Patients with a lower e’ pre-induction value or a higher E/e’ pre-induction ratio were more likely to have an improved post-induction diastolic function grade.

**Conclusion:**

The induction of general anesthesia with propofol has a significant effect on the assessment of diastolic function. Propofol may either improve or worsen the diastolic function grading. The changes in diastolic grading may be related to the pre-induction e’ and E/e’ values.

**Trial registration:**

The study was registered on the clinicaltrial.gov website (NCT04177225).

## Background

Diastolic dysfunction (DD) refers to any abnormality that causes a prolonged, delayed, or incomplete ventricular relaxation [1]. The incidence of left ventricular diastolic dysfunction (LVDD) has increased in the general patient population with estimates of 27% [2]. LVDD is strongly associated with peri-operative complications and post-operative adverse outcomes in both non-cardiac and cardiac surgical patients [3, 4]. Non-cardiac surgical patients with LVDD are found to have higher rates of both short- and long-term all-cause mortality, major adverse cardiac events (MACES), acute myocardial infarction, post-operative pulmonary edema, congestive heart failure, and prolonged hospital stay [3, 5]. Cardiac surgical patients with LVDD have more difficulty weaning from cardiopulmonary bypass and have higher in-patient cardiac deaths independent of left ventricular ejection fraction compared to those without LVDD [6, 7].

Echocardiography is key to the assessment of diastolic function. However, most patients with LVDD are asymptomatic for years before progressing to symptomatic diastolic heart failure, and therefore any degree of diastolic dysfunction is usually not identified before undergoing surgery [8, 9]. Impaired ventricular relaxation results in different physiological responses to intra-operative interventions [3], thus modifying the management of these patients. It is therefore important to identify those with impaired diastolic function to optimize their intra-operative and post-operative care. Given the potential opportunity for this diagnosis to improve peri-operative patient care the question arises as to whether it is beneficial to delay surgery to investigate diastolic function or if this assessment can be made intra-operatively, after the induction of general anesthesia. Previous studies investigating the effects of general anesthesia on diastolic function have resulted in inconsistent conclusions with respect to propofol ranging from worsening function [10, 11], to no effect [12–14] or improved function [15, 16]. This study compared assessments of diastolic function before and after the administration of propofol to determine whether induction of general anesthesia and mechanical ventilation cause a significant change in diastolic functional grading. We hypothesized that the echocardiographic assessment of diastolic function would be unaffected by propofol administration.

## Methods

This was a retrospective analysis of a subset of data from an investigator-initiated, prospective, observational, single-center study that was conducted by the Department of Anesthesiology and Pain Medicine of the University of California at Davis. The study was approved by the Institutional Review Board and registered on the clinicaltrial.gov website (NCT04177225). Written informed consent for the primary study was provided by all patients prior to surgery. Patients were at least 18 years of age, undergoing elective surgical procedures requiring general anesthesia with mechanical ventilation and invasive arterial pressure monitoring as a planned part of the intra-operative management. Exclusion criteria included: patients unable to provide informed consent, atrial fibrillation, significant arrhythmias, emergency procedures, open thoracic procedures, presence of an intra-aortic balloon pump, hemodynamic instability requiring vasopressor and/or inotropic infusions, BMI < 20 or > 40, pregnancy, and patients who were prisoners.

After written, informed consent was provided, patients were administered routine premedication (midazolam, 1–2 mg IV) and then transferred to the operating room where all American Society of Anesthesiologists (ASA) standard monitors were applied. Patients then underwent a focused transthoracic echocardiographic (TTE) examination to assess left ventricular (LV) diastolic function using the following parameters: lateral mitral annular tissue doppler velocity (e’), peak early mitral flow velocity (E) and peak late mitral flow velocity (A). General anesthesia was then induced at the discretion of the primary anesthesia care team using either propofol or etomidate followed by rocuronium. Five minutes after the induction of general anesthesia, endotracheal intubation and initiation of mechanical ventilation, the focused TTE exam was repeated measuring the same parameters. After which, anesthesia and surgery proceeded per routine.

All TTE studies were performed by one of two co-investigators, both of whom were experienced sonographers and Advanced Perioperative Transesophageal Echocardiography certified. The ultrasound examinations were recorded using a Philips EPIC iE33 ultrasound system and an X5-1 probe (Philips Healthcare, Bothell, Washington). Post-induction measurements and analysis for each study were completed by the same investigator that performed the pre-induction TTE exam.

**Fig. 1:**
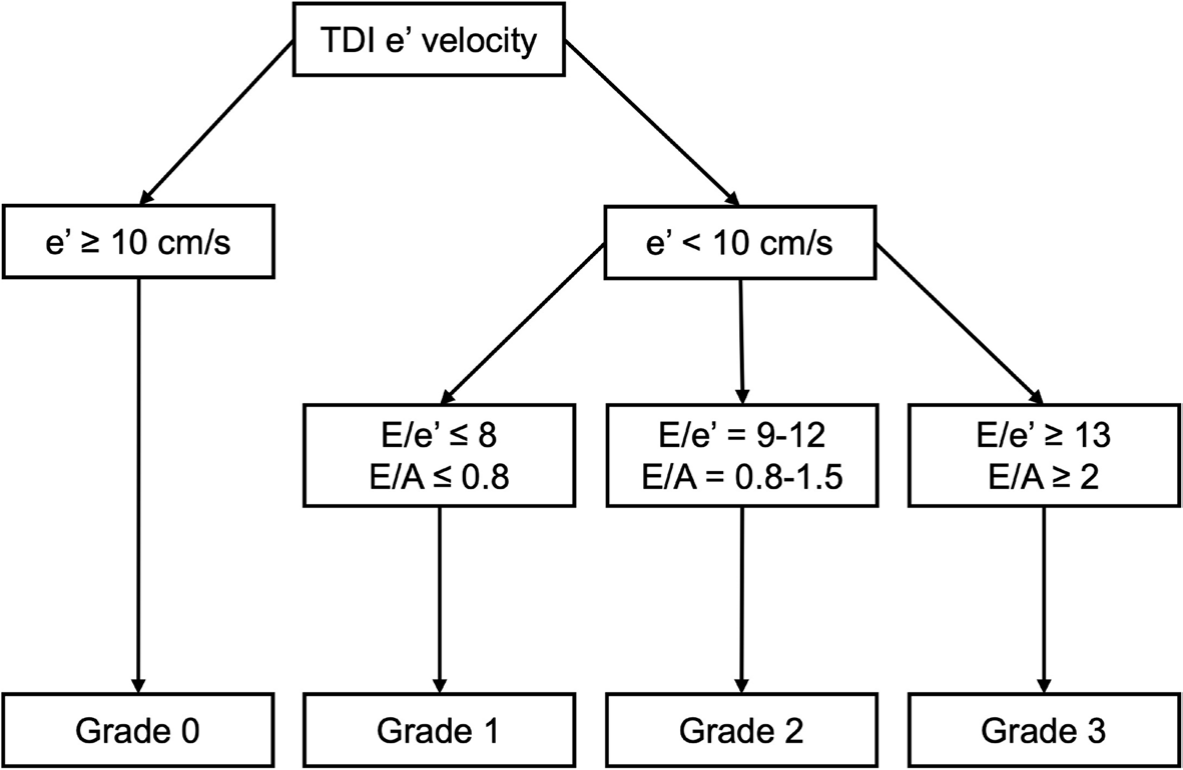
Assessment of diastolic function. Simplified algorithm for the assessment of diastolic function adapted from Swaminathan et al. [17] TDI – tissue Doppler imaging. e’ – lateral mitral annular early diastolic tissue velocity. E – Early trans-mitral filling velocity (cm/s). A - peak late mitral flow velocity (cm/s)

The diastolic function grading followed the simplified algorithm described by Swaminathan et al. 2011 (Fig. 1) [17]. Grading was based on the e’ velocity (either < 10 cm/s or $\:\ge\:$ 10 cm/s) and the mitral flow velocity ratios. If e’ ≥ 10 cm/s then grade = 0 (normal). If e’ < 10 cm/s, then further categorized as E/e’ ≤ 8 (Grade 1, Impaired Relaxation), E/e’ = 9–12 (Grade 2, Pseudonormal), and E/e’ ≥ 13 (Grade 3, Restrictive).

### Statistical analysis

All continuous data are presented as either mean ± SD or median [95% CI] for normally or non-normally distributed values, respectively. Categorical variables are presented as a number. The distribution of all continuous measurements was assessed for normality using the D’Agostino and Pearson test. The Wilcoxon matched-pairs signed rank test was used for comparisons of data that were not normally distributed. Comparisons of categorical variables were made using Fischer’s exact test. For comparison of unpaired data among multiple groups of patients, the Kruskal-Wallis test was used followed by Dunn’s Multiple Comparison test. A value of *p* < 0.05 was considered to be significant. All statistical analysis was performed using GraphPad Prism (version 10.0.0 for Windows, GraphPad Software, Boston, Massachusetts USA, www.graphpad.com).

## Results

Written, informed consent was provided by 120 patients undergoing non-cardiac surgical procedures. Seven patients were removed from further study because of atrial fibrillation (2), lack of arterial line monitoring (2), induction agents other than propofol used (2) or failure of data collection (1). The remaining 113 patients had various non-cardiac surgical procedures: vascular (58), otolaryngology (22), gastrointestinal (14), general (10), urologic (8), and general thoracic (1). There were 78 males (69.2%) and 35 females (30.8%) with an average age of 69 ± 11years, and an average weight of 79 ± 16 kg. The average induction dose of propofol was 1.7 ± 0.7 mg/kg. In the study group, 5 patients were classified as ASA physical status 2, 89 were ASA 3, and 18 were ASA 4.

**Fig. 2:**
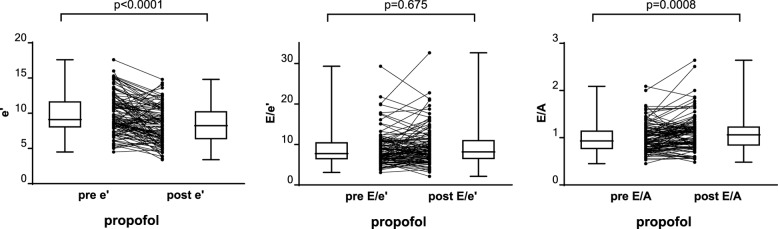
Group and individual changes for echocardiographic measurements following induction of anesthesia. e’ – lateral mitral annular early diastolic tissue velocity. E – Early trans-mitral filling velocity. A - peak late mitral flow velocity

Echocardiographic measurements are summarized in Table 1. Because most measurements were not normally distributed, all groups are presented as median [95%CI]. Pre-induction, the mitral annular tissue velocity (e’) was 9.1 [8.7, 10.5] cm/s, decreasing significantly to 8.2 [7.2, 8.9] cm/s following induction. Similarly, the early mitral filling velocity (E) decreased significantly from 79.8 [76.0, 83.4] cm/s to 66.0 [62.8, 72.0] cm/s and the late mitral filling velocity (A) decreased significantly from 82.2 [75.3, 89.0] cm/s to 64.1 [60.2, 69.0] cm/s. Individual changes for the variables used to determine diastolic functional grading are summarized in Table 1; Fig. 2.

**Table 1:** Echocardiographic measurements. e’ – lateral mitral annular early diastolic tissue velocity, E – Early trans-mitral filling velocity, A - peak late mitral flow velocity. Values of p for Wilcoxon matched-pairs sign ranked test

	Pre-induction (median [95%CI])	Post-induction (median [95%CI])	Difference (median [95%CI]) (*p*)
e’ (cm/s)	9.1 [8.7, 10.5]	8.2 [7.2, 8.9]	−1.6 [−2.1, −1.0] < 0.0001
E (cm/s)	79.8 [76.0, 83.4]	66.0 [62.8, 72.0]	−12.0[−15.0, −7.8] < 0.0001
A (cm/s)	82.2 [75.3, 89.0]	64.1 ([60.2, 69.0]	−17.0 [−22.0, −11.0] < 0.0001
E/e’	7.7 [7.1, 9.0]	8.2 [7.5, 8.9]	−0.01 [−0.44, 0.59] 0.68
E/A	0.93 [0.87, 1.04]	1.06 [0.95, 1.15]	0.07 [0.00, 0.13] 0.0008

Prior to the induction of general anesthesia, the diastolic function grade was as follows: 47 patients grade 0, 24 patients grade 1, 29 patients grade 2 and 13 patients grade 3.

**Fig. 3:**
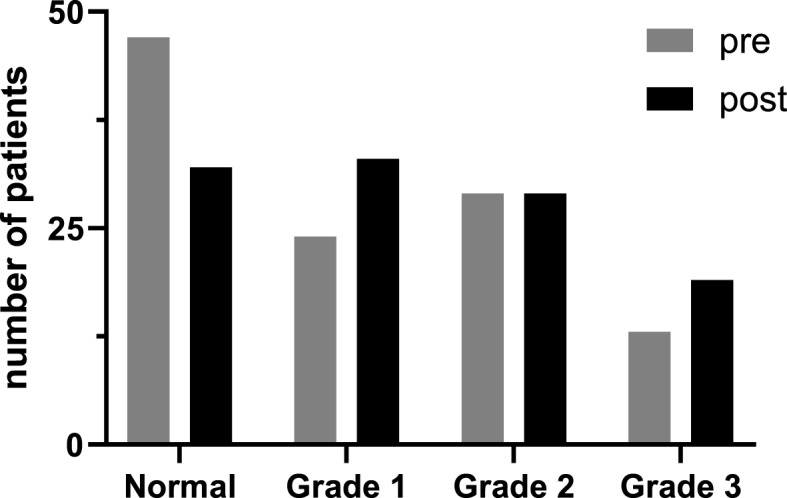
Overall changes in diastolic function grading following induction

**Fig. 4:**
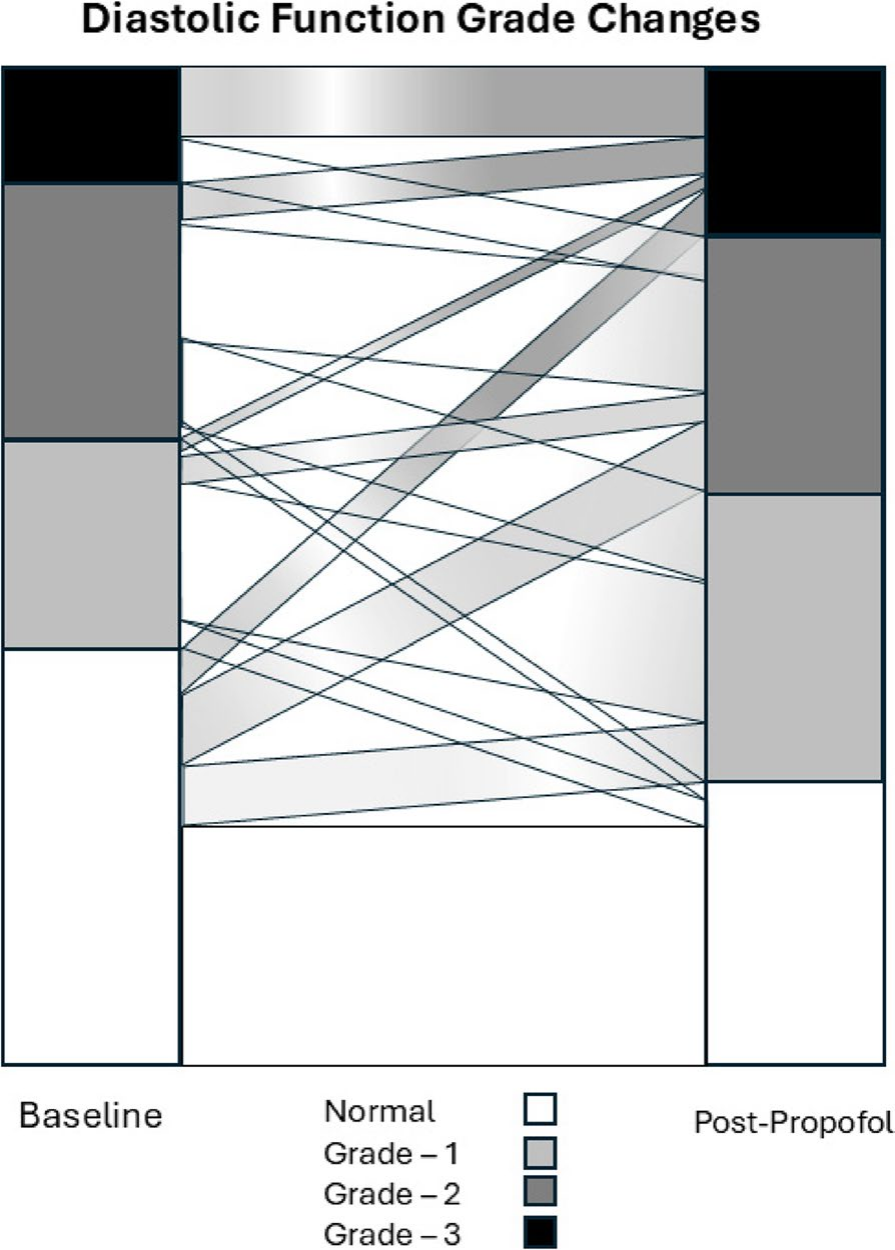
Alluvial plot of changes within each baseline functional class following induction

The overall changes in diastolic function grade are summarized in Fig. 3. Of the 113 patients, 20 (17.8%) patients had an improved diastolic function assessment (decreased grade) following propofol, 64 patients (56.6%) had the same diastolic function grade post-induction, while 29 (25.6%) patients had worsening diastolic function (increased grade). The individual changes within each group are presented in Fig. 4. The demographic and echocardiographic data for each of these 3 groups (decreased grade [improved function], no change, increased grade [decreased function], are summarized in Table 2.

All patients were subsequently divided into three groups based upon their change in diastolic function assessment: decreased grade (improved function), no change, or increased grade (worse function). Comparisons of their demographic and pre-induction echocardiographic values were made to evaluate possible characteristics associated with the observed changes. These results are summarized in Table 2.

**Table 2:** Comparison of demographic and pre-induction echocardiographic values in patients grouped according to subsequent post-induction changes in diastolic function grading. “*” indicates difference from patients with a lower functional grade (improved function). “#” indicates difference from patients with no change in functional grade

	Decreased Grade (improved function)	No Change	Increased Grade (worse function)
Age (years)	72 ± 9.8	67 ± 12	74 ± 7 # *p* = 0.04
Sex (M/F)	13/7	43/21	22/7
Weight (kg)	77 ± 15	80 ± 16	79 ± 17
ASA grade			
2	0	5	0
3	18	49	22
4	2	10	7
e’ (cm/s)	7.9 [6.6, 8.6] # *p* = 0.0009	9.5 [0.9, 1.1]	11.5 [9.5, 12.3] * *p* < 0.0001
E (cm/s)	80 [71, 88]	80 [75, 88]	77 [67, 91]
A (cm/s)	92 [67, 102]	81 [69, 86]	86 [70, 107]
E/e’	10.0 [9.0, 12.0] # *p* = 0.01	7.3 [6.6, 9.1]	6.9 [6.4, 8.7] * *p* = 0.002
E/A	0.87 [0.74, 1.00]	1.00 [0.92, 1.10]	0.85 [0.73, 1.00]

## Discussion

The induction of general anesthesia with propofol is associated with significant decreases in lateral mitral annular tissue doppler velocity, peak early, and peak late mitral flow velocity. Consequently, 43% of the patient’s studies had an associated change in their diastolic function grades. Of those patients, 41% had an improvement, while 59% had a worsening of their diastolic function grade. Patients with a lower e’ pre-induction value or a higher E/e’ pre-induction ratio were more likely to have an improved post-induction diastolic function grade.

Left ventricular heart failure can be categorized as either systolic or diastolic [9, 18]. Diastolic heart failure, also known as heart failure with preserved ejection fraction (HFpEF) greater than 50%, accounts for approximately 50–60% of heart failure cases in the community and 40–50% of incident heart failure overall [18]. HFpEF has a mortality rate of 394 per 10,000 per years [19]. HFpEF is a clinical diagnosis based upon symptoms and pathology. Independent of this diagnosis, patients with left ventricular diastolic dysfunction have higher rates of peri-operative and post-operative complications when compared to surgical patients with normal diastolic function [3–5]. This background emphasizes the importance of diagnosing LVDD in the non-cardiac surgical population to optimize their peri-operative management. Despite the potential clinical management ramifications, only 26 (23%) of the 113 patients in this study had a recent, pre-hospitalization cardiac ultrasound examination. Eleven (42%) of these patients had normal left ventricular diastolic function.

Diastolic function can be assessed and stratified into grades using TTE [20]. For our study, we used the simplified algorithm described by Swaminathan et al. [17] All 3 of the primary echocardiographic measurements for this assessment (e’, E and A) decreased significantly after the induction of general anesthesia with propofol. Consequently, of 113 total patients, 49 patients (43%) had a change in their diastolic function grading from pre-induction to post-induction. Out of those 49 patients, 20 (17.8%) had an improved diastolic function grade, while 29 (25.6%) had a worsening diastolic function grade. To examine the correlations between intra-operative and pre-operative assessments we reviewed the 26 patients in this study who had an echocardiographic examination prior to their hospitalization. The sample size was too small for a formal sub-group analysis. In 16 (62%) patients the baseline assessment was identical to the pre-hospitalization assessment. Six of these patients had some degree of LVDD, in 3 of these patients the functional grade worsened after propofol. Four patients in this group with normal function at baseline developed LVDD after propofol. Of the remaining 10 patients, 5 with a pre-hospitalization diagnosis of LVDD had normal function at baseline. Four of these patients demonstrated more severe LVDD (grade 2 or 3) after receiving propofol, the other remained normal. For all the remaining 5 patients, the diastolic functional grading at baseline was more severe than the pre-hospitalization assessment. Three of these patients were unchanged after propofol, one had an increased grade, the other decreased. This scatter suggests it may be difficult to predict intraoperative diastolic function based upon a pre-hospitalization examination.

Given the variable changes observed following propofol administration, the demographic and echocardiographic characteristics of the patients were examined to evaluate the potential for predicting the observed changes in diastolic function grading. Statistically, patients who had worse functional grading after propofol were older (74 ± 7 years) than those with no change (67 ± 12 years), but this difference would not be considered clinically significant as those patients with improved functional grading were of comparable age (72 ± 9.8 years). However, patients who had a lower e’ pre-induction value (7.9 [6.6, 8.6] cm/s) or a higher E/e’ pre-induction value (10.0 [9.0, 12.0]) (worse diastolic function) were more likely to have an improved post-induction grade, while patients who had a higher e’ pre-induction value (11.5 [9.5, 12.3] cm/s) or a lower E/e’ pre-induction value (6.9 [6.4, 8.7]) (better diastolic function) were more likely to have a worse post-induction grade. In conclusion, propofol may either improve or worsen diastolic function and the overall change might be related to patients’ pre-induction echocardiographic parameters. It cannot be assumed that it will consistently worsen.

Some limitations of this study should be highlighted. The impact of propofol was not the primary outcome of this clinical trial. As such, the patient population, drug dose and administration and assessment sequence were not optimized to specifically evaluate the focus of this analysis. The study did not focus on patients presenting with a diagnosis of DD. Similarly, the study did not focus on patients with a diagnosis of HFpEF as no data was collected to characterize systolic left ventricular function. The dose of propofol was not standardized or randomly varied. The post-induction TTE was performed five minutes after induction of general anesthesia, onset of neuromuscular paralysis and initiation of mechanical ventilation. Some of these factors could influence the assessment of diastolic function in addition to propofol. In addition, our study did not analyze the changes in diastolic function at other time points such as further after induction or following administration of other drugs. Lastly, we utilized a simplified algorithm for the assessment of diastolic function that has been demonstrated to have increased utility in the OR clinical setting and be clinically predictive for MACE [17]. The comprehensive guidelines for the assessment of left ventricular diastolic function from the American Society of Echo cardiography include measurements of both lateral and septal e’, tricuspid regurgitation jet velocity and the left atrial volume index [20]. These measurements may have provided additional information but are difficult to collect expeditiously in the OR clinical setting.

In conclusion, when comparing pre-induction and post-induction data, our results indicate that induction with propofol has a significant effect on diastolic function and can lead to either improvements or exacerbations of diastolic function grading. Moreover, our study showed that whether a patient’s diastolic function improves or worsens with propofol may be related to their pre-induction cardiac function as assessed by the echocardiographic e’ and E/e’ parameters. Diastolic function should be assessed prior to the induction of general anesthesia to guide the peri-operative care of non-cardiac surgical patients. The effects of propofol on diastolic function (better or worse) might provide additional guidance for peri-operative management and merit further investigation.

## Data Availability

The data sets used and/or analyzed during the current study are available from the corresponding author on reasonable request.

## Abbreviations

A: Peak late mitral flow velocity
ASA: American Society of Anesthesiologists
DD: Diastolic dysfunction
e’: Mitral annular tissue doppler velocity
E: Peak Early Mitral Flow Velocity
HFpEF: Heart Failure with preserved Ejection Fraction
LVDD: Left ventricular diastolic dysfunction
MACE: Major adverse cardiac events
TDI: Tissue doppler imaging
TEE: Transesophageal echo
